# Novel mutation G324C in *WNT1* mapped in a large Pakistani family with severe recessively inherited *Osteogenesis Imperfecta*

**DOI:** 10.1186/s12929-018-0481-x

**Published:** 2018-11-17

**Authors:** Mehran Kausar, Saima Siddiqi, Muhammad Yaqoob, Sajid Mansoor, Outi Makitie, Asif Mir, Chiea Chuen Khor, Jia Nee Foo, Mariam Anees

**Affiliations:** 1 Department of Biochemistry, Faculty of Biological Sciences, Quaid-i-Azam University, University Road, Islamabad, Post code 45320 Pakistan; 2 Institute of Biomedical and Genetic Engineering (IBGE) Islamabad, Islamabad, 44000 Pakistan; 3 Department of Genetics, Children Hospital, Lahore, Pakistan; 4 Atta-ur-Rehman School of Applied Biosciences, NUST, Islamabad, Pakistan; 5 Children’s Hospital, University of Helsinki and Helsinki University Hospital, Helsinki, Finland; 6 Folkhälsan Institute of Genetics, Helsinki, Finland; 7 Department of Bioinformatics & Biotechnology, Faculty of Basic and Applied Sciences, International Islamic University (IIU), H-10, Islamabad, 44000 Pakistan; 8 Human Genetics, Genome Institute of Singapore, A*STAR, Singapore, 138672 Singapore; 9 Lee Kong Chian School of Medicine, Nanyang Technological University, Singapore, 308232 Singapore; 10 Department of Microbiology, Faculty of Life Sciences, University of Central Punjab, Lahore, Pakistan

**Keywords:** WNT signaling, Whole-exome sequencing, Osteoporosis, Osteogenesis imperfecta

## Abstract

**Introduction:**

*Osteogenesis imperfecta* (OI) is a clinically and genetically heterogeneous disease with skeletal fragility and variable extra-skeletal manifestations. To date several point mutations in 18 different genes causing different types of OI have been identified. Mutations in *WNT1* compromise activity of the osteoblasts leading to disturbed bone mass accrual, fragility fractures and progressive skeletal abnormalities. The present study was conducted to determine the underlying genetic cause of an autosomal recessive skeletal dysplasia in a large consanguineous family from Chinute, Pakistan.

**Materials and methods:**

Blood was collected from 24 individuals of affected family along with clinical data. Homozygosity mapping was performed to confirm consanguinity. SNPs were identified, followed by whole exome and Sanger sequencing. In silico characterization of WNT1 mutation was performed using multiple platforms.

**Results:**

Nine affected family members exhibited severe bone deformities, recurrent fractures, short stature and low bone mineral density. SNP array data revealed homozygous segments > 1 Mb in length accounting for 2.1–12.7% of the genome in affected individuals and their siblings and a single 6,344,821 bp homozygous region in all affected individuals on chromosome 12q12-q13. This region includes two potential OI candidate genes *WNT1* and *VDR*. We did whole-exome sequencing for both genes in two patients and identified a novel damaging missense mutation in exon 4 of *WNT1*: c.1168G > T *(*NM_005430) resulting in p.G324C. Sanger sequencing confirmed segregation of mutation with the disease in family.

**Conclusion:**

We report a novel mutation responsible for OI and our investigation expands the spectrum of disease-causing *WNT1* mutations and the resulting OI phenotypes.

**Electronic supplementary material:**

The online version of this article (10.1186/s12929-018-0481-x) contains supplementary material, which is available to authorized users.

## Introduction

Osteogenesis imperfecta (OI) is a genetic disorder with severe bone abnormalities and is the most common form of primary osteoporosis in children [1]. Clinical phenotype may vary from prenatal lethal forms to milder forms with only a few fractures during life time. Severely deforming forms may have lifelong bone fractures along with visible skeletal deformities, short stature, dentinogenesis imperfecta, blue sclerae, and hearing loss [2]. The incidence of OI is approximately 1 in 15,000–20,000 live births and most cases (~ 90%) follow an autosomal dominant pattern of inheritance with mutations in either *COL1A1*(MIM 120150) or *COL1A2* (MIM 120160) [1]. Rest of the 10% cases follow the autosomal recessive pattern of inheritance with mutations in *FKBP10*, *P3H1*, *LEPRE1, PLOD2, PPIB, SERPINF1, SERPINH1, SP7, BMP1*, *TMEM38B, CRTAP, CREB3L1, IFITM5* and/or *WNT1*. LRP5 gene may follow any of the autosomal dominant or recessive patterns; PLS3 gene follows x-linked mode of inheritance [3–7]. Most of these genetic defects induce a qualitative or quantitative change in type I collagen, the main structural protein in the skeleton. Impaired bone-matrix formation and mineralization ultimately devastate the skeletal flexibility and integrity [8]. *WNT1* (MIM 164820) has an integral role in osteoblast differentiation and is involved in the regulation of the WNT induced beta-catenin signaling pathway. Biallelic defects in *WNT1* result in decreased expression of type I collagen [1]. Mutations encoding other components of the signaling pathway have been reported also in other disorders with abnormal bone mass, suggesting that the pathway has an integral role in the regulation of bone homeostasis [8].Only a few cases of OI caused by *WNT1* mutations have been described thus far and the spectrum of clinical manifestations remains inadequately characterized. Here we present a large consanguineous Pakistani family in which several family members present with severe deforming OI caused by a novel homozygous mutation in *WNT1*. Wnt1-related osteogenesis imperfecta has symptoms including scoliosis, developmental delay and short stature. WNT1 (Wnt Family Member 1) is an important gene associated with Osteogenesis Imperfecta.

## Material and methods

### Subjects

We identified a large consanguineous Pakistani family with multiple family members affected by severe bone deforming OI. Blood samples for genetic analyses were collected from all available family members after informed consent. A pedigree was constructed to draw the relationships and the disease status.

### Ethics, consent and permission to publish

This study was approved by the institutional review board of the Institute of Biomedical and Genetic Engineering (IBGE), Islamabad, Pakistan. All adult participants gave written informed consent to participate in this study. For minors, consent was obtained from their legal guardians / parents. Permission was also obtained from the patients to use their data and pictures for publishing.

### Phenotypic data

Clinical examination of patients was performed at the Indoor Patient Department (IPD) of Children’s Hospital and radiography was done at Radiology Department of Children’s Hospital, Lahore, Pakistan.

### Genotyping

Blood samples were drawn from 24 individuals from the affected family that included affected (*n* = 9)and unaffected(*n* = 15) individuals. Genomic DNA was extracted using standard Sambrook protocol [9]. Quality of DNA was assessed by using Qubit dsDNA HS (High Sensitivity) Assay kit (Invitrogen) and 24 samples were genotyped on the IlluminaOmniExpress 24v1–0-A BeadChip array for a total of 716,503 genetic markers. After removal of single nucleotide polymorphisms (SNPs) that were monomorphic orfailed genotyping in > 1 sample, 392,260 genome-wide SNP markers remained for further analysis. We confirmed the reported familial relationships among genotyped samples using PLINK identity by descent (IBD) analysis (−genome) [10, 11]. We then scanned the data for large homozygous segments (> 1 Mb) which are shared among affected individuals but not for the unaffected individuals. Homozygosity mapping was conducted using PLINK v1.07 using the default settings [10].

### Whole exome sequencing (WES) and sanger sequencing

Targeted enrichment was performed on 1 μg of genomic DNA from two affected individuals (III:5 and III:15) using the Nimblegen SeqCap EZ Exome v3 kit and barcoded for sequencing on a single lane of a multiplexed 2 × 101 bp sequencing run on the Illumina HiSeq 2000platform. Each individual was sequenced to a mean coverage of 45.2–46.7 reads per target base, with 97% of the target exome covered by 10 or more reads. Reads were mapped using BWA v1.7 [12] and variants were called using the GATK v2 Unified Genotyper following the recommended guidelines by GATK ‘Best practices for variant calling v3’ [13]. We used the following primer sequences for the segregation of detected mutations in *WNT1* by polymerase chain reaction and Sanger sequencing: WNT1-ForwardGAAATCGCCCAACTTCTGCA and WNT1-Reverse AGTGCTAGCGAGTCTGTTTGG.

### In silico predictions and validation of novel WNT1 mutation G324C

I-Tasser [14, 15] was used for three dimensional (3D) model predictions. Protein 3D models reliability was checked using RAMPAGE server [16]. STITCH4 database [17] was used to predict functional protein partners. Pockets on three-dimensional structures of proteins were identified using CASTp server [18, 19]. Meta SNP [20] and I-Mutant2.0, were utilized to estimate effect of mutation on stability of protein and to determine whether the mutation has an impact on normal function of protein or not. Docking analysis was carried out using PatchDock server [21]. The refinement of first 10 docked complexes obtained through PatchDock was done using FireDock [22, 23]. Representations (2-Dimensional) and analysis of protein-ligand interaction complexes was done using LIGPLOT [24]. Meta SNP was also utilized to analyze effect of mutation on protein.

## Results

### OI phenotype examination

**Fig. 1:**
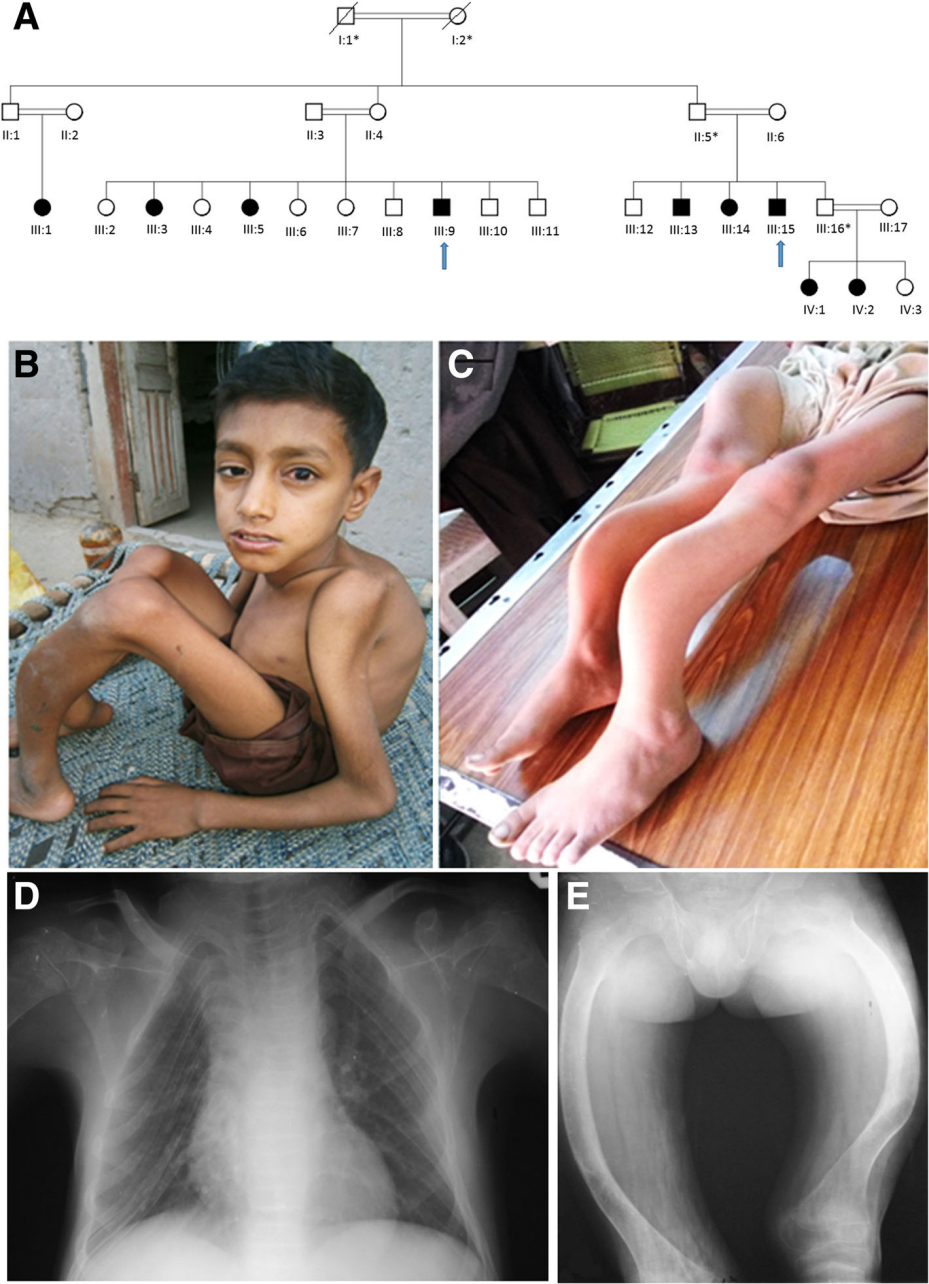
**a**) Pedigree of the family with osteogenesis imperfecta. Affected individuals are marked with black symbols, arrows indicate patients whose phenotypes are described in the text. Asterisks indicate individuals whose DNA was not available for the study. II:1 and II:2 are estimated to be first cousins (IBD proportion ~ 11%). III:3 and III:6 are half-siblings (IBD proportion ~ 21%). **b**) Patient 1 at age 11 years with severe short stature, short neck, small thorax and long-bone deformities with marked anterior bowing of the bones. He had slightly blue sclerae and a mild squint. **c**) Patient 2 at 10 years was wheel-chair bound and had marked skeletal deformities with anterior angulation of the long bones in lower limbs. **d**) Radiographs of Patient 2 at 10 years showing severe osteoporosis with thin ribs, flat vertebrae, and osteopenia of the humeri. **e**) The femoral bone modeling was normal but both femurs were significantly bent

We examined a large consanguineous Pakistani family with nine affected members (Fig. 1a) diagnosed with OI type 3 on the basis of apparent clinical features and radiological findings. All affected individuals manifested similar features (Additional file 1: Table S1). The two index patients are described here in more detail.

### Patient 1

Patient 1 (III:9) was a 11 years old male, who presented with a history of multiple fractures after mild trauma since the age of 6 months. His first fracture of right clavicle occurred at the age of 6 months, followed by fracture of right tibial shaft at the age of 8 years, and fracture of right and left femora at 9 years. Pregnancy and birth were uneventful. His parents were distant cousins (IBD proportion < 6%). Presently, the index individual is wheelchair bound because of multiple fractures, deformities and weakness of lower extremities. His weight is 20 kg, height 106 cm and head circumference 52 cm. Physical examination revealed brachycephalic head, flat face, mild blueness of sclerae, right eye squint, short neck, marked thinness of upper and lower extremities, anterior angulation of both femora and right tibia, and flat feet (Fig. 1b). Skeletal radiographs revealed generalized osteopenia, bowing of clavicles, compression of thoracic vertebrae, narrowing of intercostal spaces, and bowing of long bones.

### Patient 2

Patient 2(III:15) was a 10 years old male, wheelchair bound, measuring 18 kg in weight, 107 cm in height and 51 cm in head circumference. Pregnancy and delivery were uneventful. Parents were also distant cousins (IBD proportion ~ 6%).His health remained poor since birth. He was able to walk but never became able to run. At the age of 8 years he sustained fractures of right humerus and right tibia and femur after trivial traumas (Fig. 1c). Clinical examination showed triangular face, normal teeth, right eye squint, normal sclerae, and wide protruding chest with increased antero-posterior diameter. Extremities were thin and showed bilateral mild angulation of proximal humeri and marked bowing of right femur. His feet were flat. Radiological examination showed generalized osteopenia, platyspondyli in all vertebrae, narrowing of inter-costal spaces, globular pelvis, bowing of distal parts of right tibia and fibula, and mild bowing of right radius and ulna (Fig. 1d and e).

### Molecular analysis

#### Homozygosity mapping

Homozygous segments of > 1 Mb in length accounted for 2.1–12.7% of the genome in all 9 affected individuals and their full siblings, confirming that these individuals are the offspring of consanguineous unions. We identified a single 6,344,821 base pair segment on chromosome 12q12-q13 (chr12:46,084,699-52,429,520) that was homozygous only in affected individuals (Additional file 2**:** Table S2). Two candidate genes for bone density were located in the homozygous segment, the vitamin D receptor *VDR* and the wingless-type integration site family, member 1 *WNT1.*

#### Whole exome sequencing and segregation analysis

For identification of causal mutation, we performed whole exome sequencing analysis in two affected cousins III:5 and III:15 **(**Additional file 3: Table S3). A total of 43,494 variants were found in these two individuals, out of which 29,745 were coding and 13,287 were nonsynonymous, frameshift or splice site variants in well-annotated transcripts. Of these, 1319 were rare, either absent or present in less than 1% of all populations in HapMap, 1000 genomes populations [8] and the NHLBI exome variant server (EVS) databases (URL: http://evs.gs.washington.edu/EVS/). Of these, 20 were homozygous in both affected cousins, 3 resided within the shared homozygous interval on chromosome 12 and only one was predicted to be damaging and not found in any of the above SNP databases or the Exome Aggregation Consortium (ExAC) database. This was identified as a novel damaging mutation in *WNT1* c.1168G > T (RefSeq NM_005430) resulting in p.Gly324Cys. We subsequently Sanger sequenced samples of 24 members of the family and verified that this mutation segregated perfectly with the disease (Additional file 4**:** Figure S1). This mutation has not been reported previously in any of the publications or online databases including ‘ClinVar’, ‘HGMD(R)’ and ‘ExAC’. No pathogenic mutations were found in CCNT1 or VDR genes; there was only a synonymous SNP in VDR gene with no amino acid change.

#### In silico characterization of WNT1 mutation G324C

**Fig. 2:**
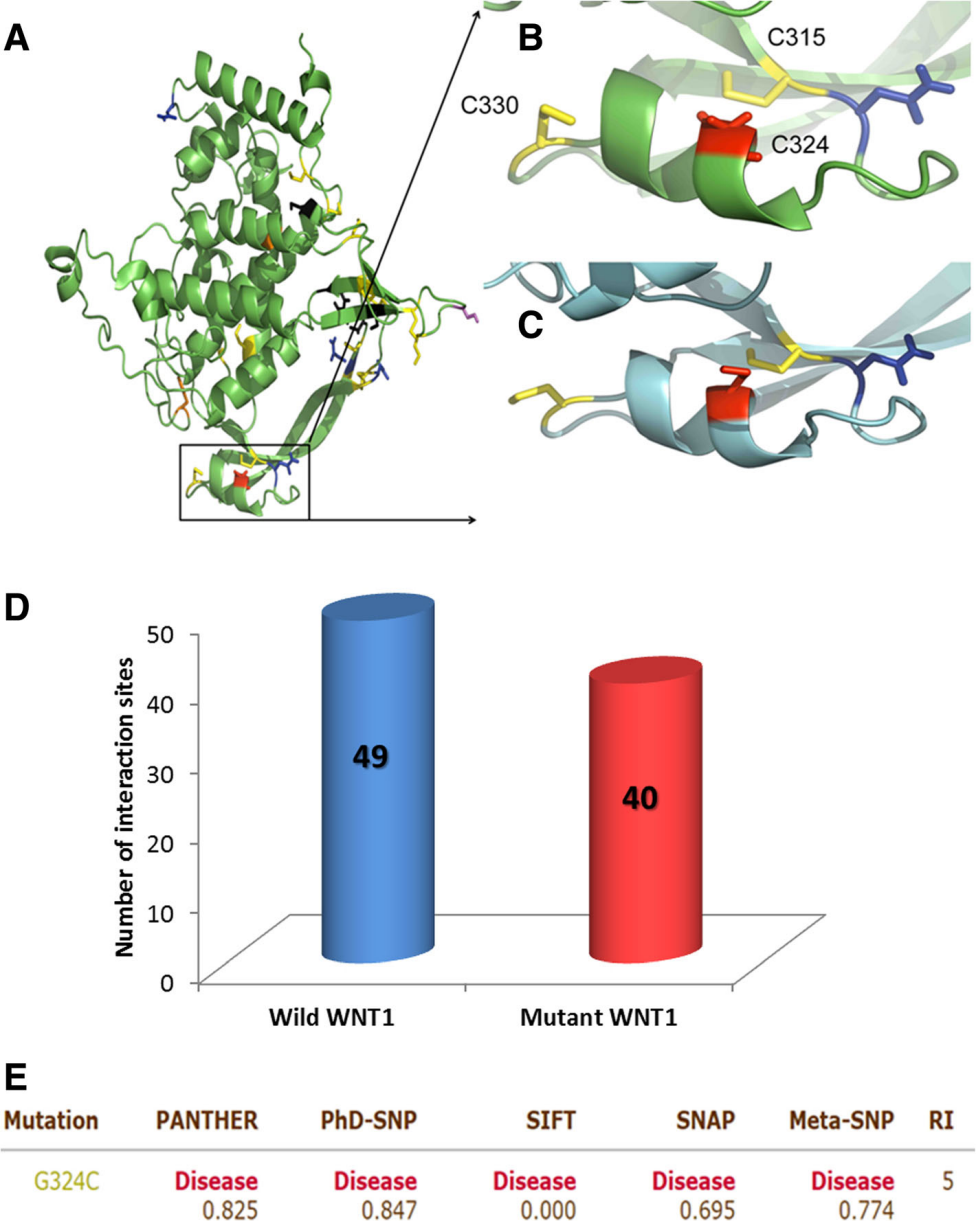
**a**) WNT1 protein’s functional residues that are glycosylated are colored blue while the residues forming disulfide linkages are in yellow and the natural variants (known mutations) are shown in black. Mutation G to C at position 324 is shown in red. **b**) Wild type zoomed in at the mutation site, the red residue is the G324 and the yellow residues to its right and left are C315 and C330 respectively. The blue residue at the side is the N316 which is glycosylated. **c**) Same site for the mutated protein. **d**) Comparison of interaction sites for WNT1 (wild and mutant forms). **e**) Meta SNP prediction for WNT1 mutation (p.G324C) effect

To gain insight into the structural implications of the mutation p.G324C, we modelled the wild type and mutant proteins using the online I-Tasser server (Fig. 2a, b and c) [9]. Models with the best c-score were selected for further analysis. Interaction sites as predicted by the CASTp results showed a decrease in available pockets in case of mutant compared to the wild type, 40 compared to 49, respectively; clearly indicating the effect of mutation upon the binding capacity of the protein (Fig. 2d). Meta SNP hires with it different predictors (PATHER, PhD-SNP, SIFT and SNAP) to calculate mutation impact on normal protein. Value reported under each prediction is given in (Fig. 2e). All predictors show it as disease causing which validate our result. Obtained calculated values are interpreted as following:

- PANTHER: Between 0 and 1. (If > 0.5 mutation is predicted Disease)
- PhD-SNP: Between 0 and 1. (If > 0.5 mutation is predicted Disease)
- SIFT: Positive Value (If > 0.05 mutation is predicted Neutral)
- SNAP: Output normalized between 0 and 1 (If > 0.5 mutation is predicted Disease)
- Meta-SNP: Between 0 and 1. (If > 0.5 mutation is predicted Disease)

**Fig. 3:**
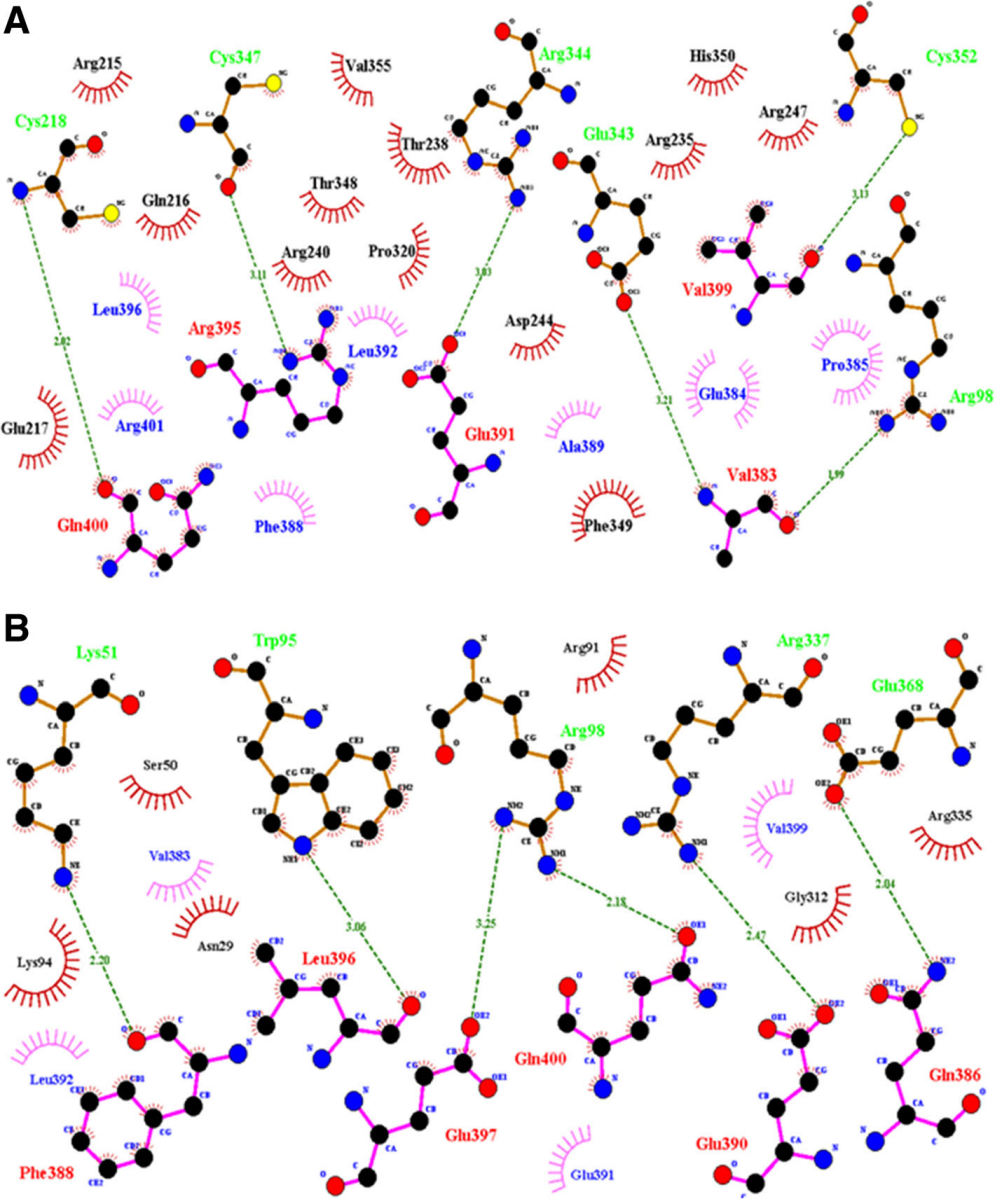
**a**) 2D Ligplot representation ofdocking interaction of normal WNT1 with AXIN1. **b**) 2D Ligplot representation ofdocking interaction of mutant WNT1 with AXIN1. Receptor residues involved in hydrophobic interactions are represented by brick red spoked arcs (), hydrogen bonding shown by green dotted lines (). Receptor residues involved in H-bonding are shown in green color. Ligand residues involved in H-bonding are shown in red color

**Fig. 4:**
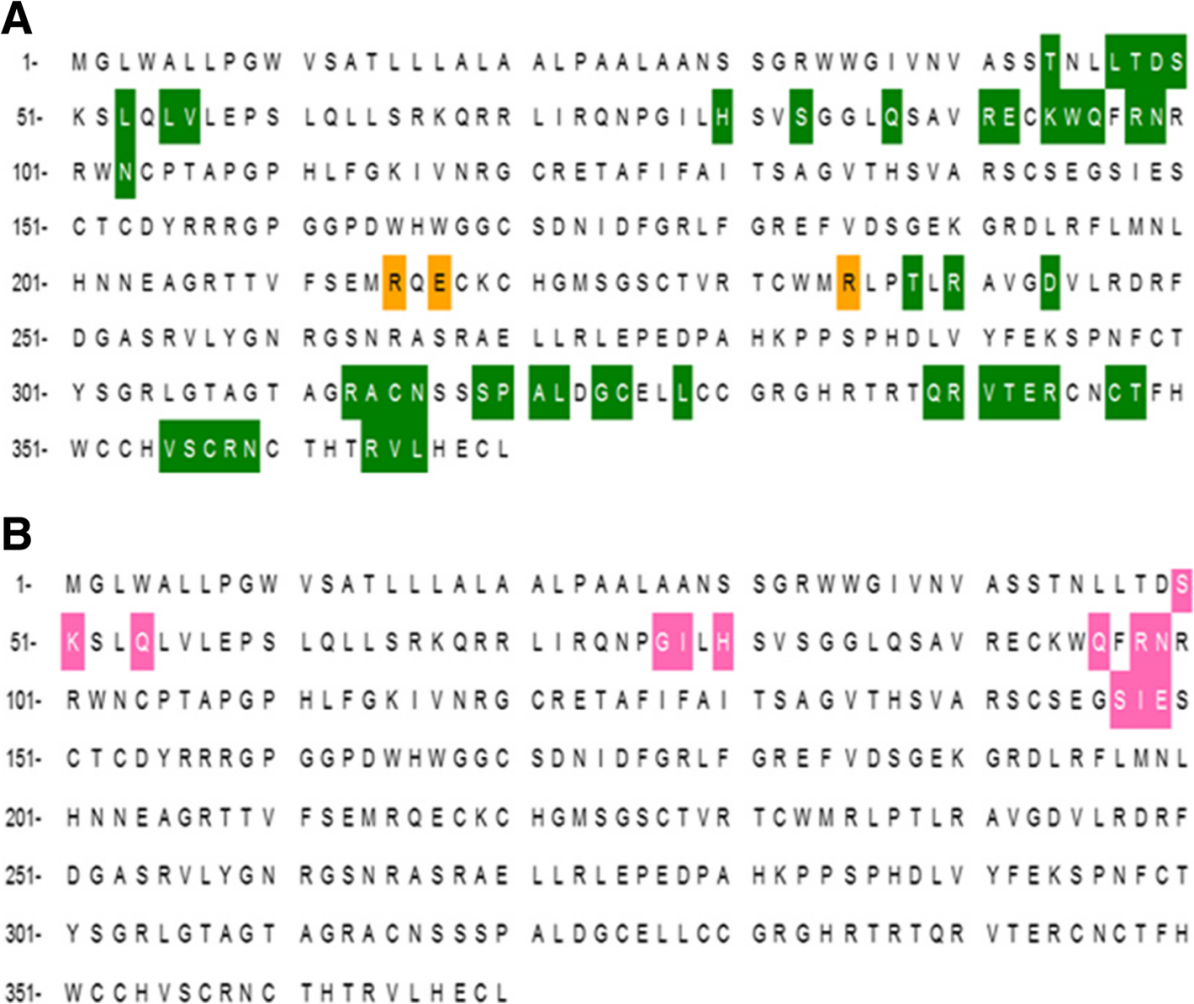
CASTp Results: Interaction sites containing residues involved in Docking **a**) Normal WNT1 vs AXIN1. **b**) Mutant WNT1 vs AXIN1

With maximum score of 0.988, Axin 1 has been selected as ligand for further analysis. It is a component of the beta-catenin destruction complex required for regulating catenin beta 1 (CTNNB1) levels through phosphorylation and ubiquitination, and modulating Wnt-signaling. It controls dorso ventral patterning via two opposing effects; down-regulates CTNNB1 to inhibit the Wnt signaling pathway and ventralize embryos, but also dorsalizes embryos by activating a Wnt- independent JNK signalling pathway. Wnt signalling probably facilitates the phosphorylation of CTNNB1 and APC by GSK3B and is likely to function as a tumor suppressor. Ligplot results for the docking interactions of wild and mutant type WNT1 with ligand AXIN 1 are shown in Fig. 3a and b, respectively. Residues involved in hydrogen bonding and hydrophobic interactions are given in Table 1. G324C mutation caused a huge impact on the normal structure and conformation of the protein molecule. This can be well observed through differences in number and position of residues involved in both types of docking interactions. This is due to the alteration of the interaction site after mutation that affected protein ligand interaction. As per CASTp predictions, pocket number 49 and 17 (Fig. 4a) contain maximum residues which are involved in docking of normal protein with ligand while pocket number 12 and 38 (Fig. 4b) contain residues involved in docking interaction of mutant protein. The analysis not only identifies active sites in the WNT1 structure but also validates the docking results.

**Table 1:** Summary of residues involved in WNT1 docking interactions (Wild & Mutant)

Receptor/Ligand	Hydrogen Bond Interactions		Hydrophobic Bond Interactions	
	Ligand Residues	Receptor Residues	Ligand Residues	Receptor Residues
Wild WNT1-Axin 1	Val383, Glu391, Arg395, Val399, Gln400	Arg98, Cys218, Arg344, Cys347, Cys352	Glu384, Pro385, Phe388, Ala389, Leu392, Leu396, Arg401	Arg215, Gln216, Glu217, Arg235, Thr238, Arg240, Asp244, Arg247, Pro320, Thr348, Phe349, His350, Val355
Mutated WNT1-Axin 1	Gln386, Phe388, Glu390, Leu396, Glu397, Gln400	Lys51, Trp95, Arg98, Arg337, Glu368	Val383, Glu391, Leu392, Val399	Asn29, Ser50, Arg91, Lys94, Gly312, Arg335

## Discussion

**Fig. 5:**
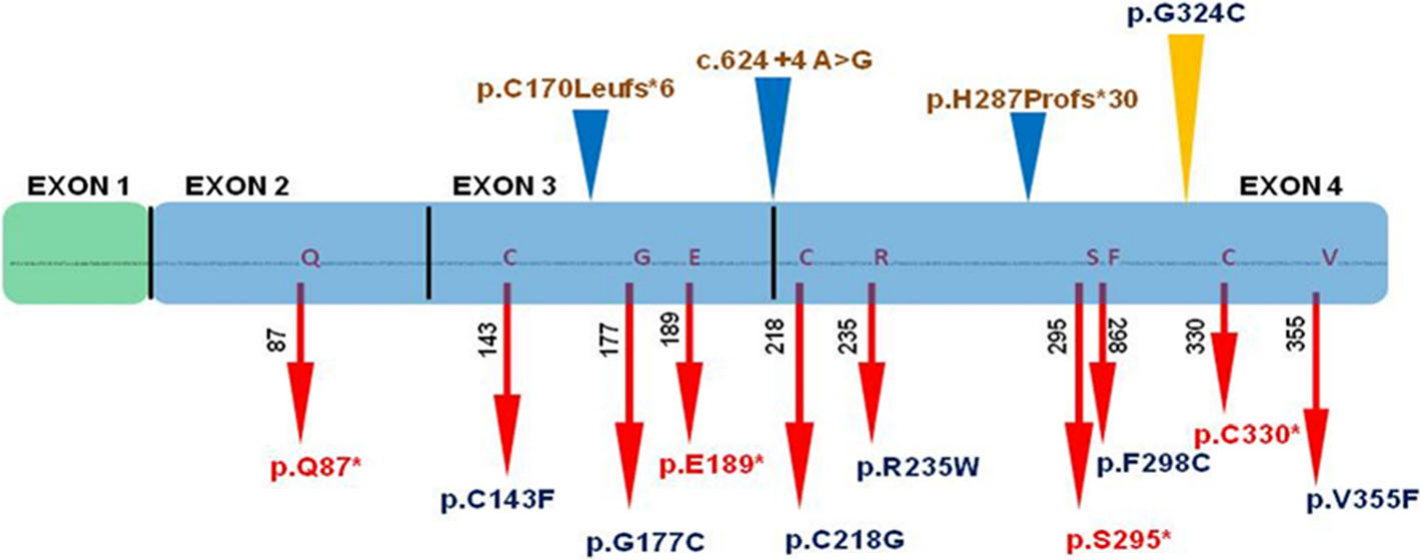
All the published WNT1 mutations associated with recessive OI. Red arrow with red text shows all the stop mutations. Red arrow with blue text point mutations. Blue wedge represents the duplication or deletions. Yellow wedge shows our novel mutation (Figure is made up to scale)

By performing homozygosity mapping and whole exome sequencing, we identified a novel homozygous mutation in *WNT1* as the cause of osteogenic imperfecta in this large consanguineous family. The novel deleterious mutation segregated with the phenotype and in the homozygous individuals resulted in severe clinical findings consistent with deforming type 3OI, thereby providing basis for genetic diagnosis of OI in the family. OI comprises multiple heterogeneous skeletal phenotypes and some forms lead to marked deformities of upper and lower limbs. To date several biallelic missense and nonsense mutations in WNT1 have been identified with multiple overlapping skeletal manifestations (Fig. 5).

**Fig. 6:**
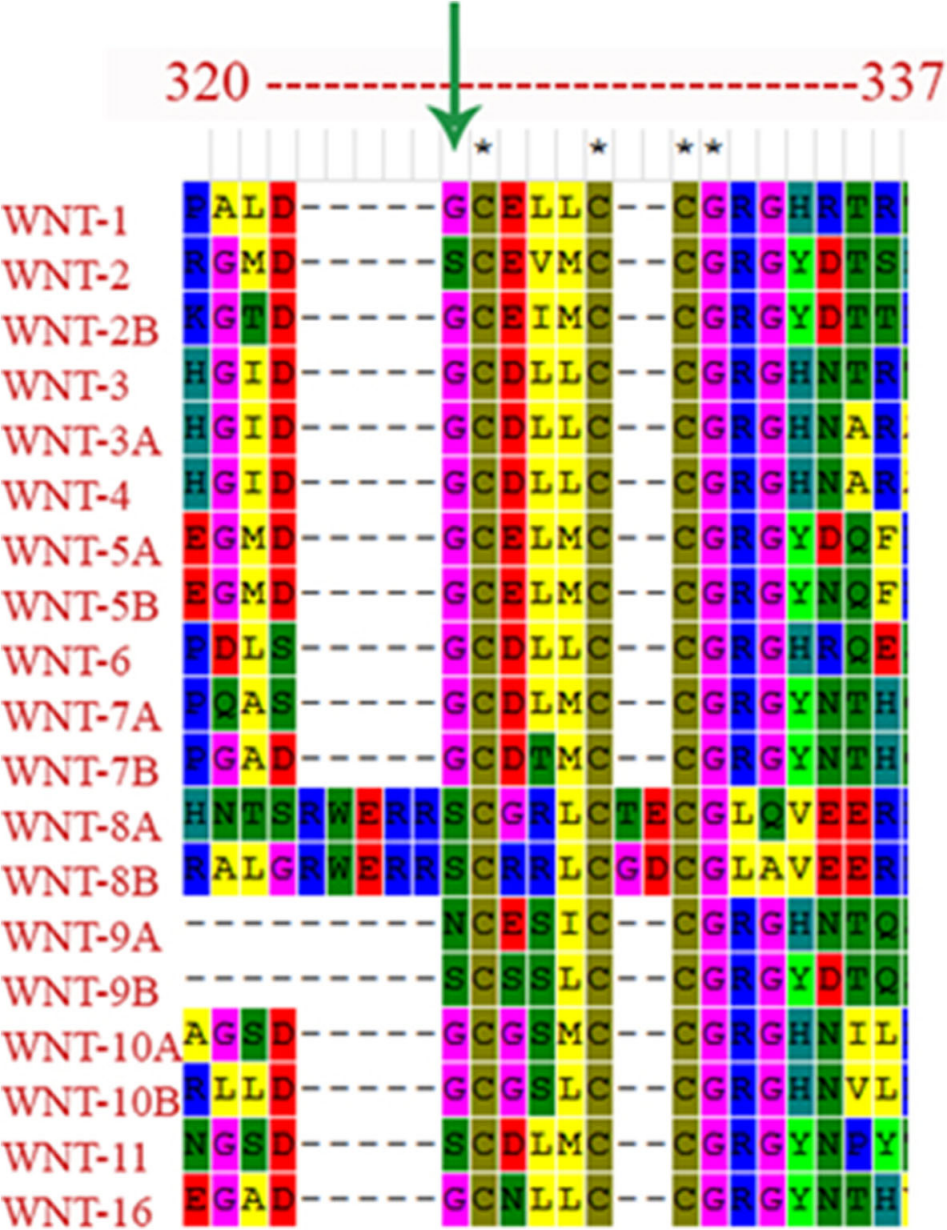
Alignment of all the WNT proteins indicating the conserved position of glycine 324 in the WNT family

The p.G324C mutation is predicted to be damaging to the WNT1 because it occurs at a highly conserved position (Fig. 6). The mutated residue (position 324) is present on a small α-helix with a number of important functional residues in the vicinity. Nearby asparagine is glycosylated while cysteine residues at 315 and 330 form a disulfide linkage. The glycosylation and the disulfide linkage are important for protein stability. Therefore, the mutation at position 324 (Gly to Cys) can be detrimental to protein stability and therefore, functionality. An adjacent cysteine residue (position 325) may well present itself for a potential disulfide linkage with the mutated cysteine residue. On the other hand the mutated cysteine may engage the cystein at 315 instead and compete for a disulfide linkage with the cysteine at 330. In either case the protein stability at this site may be effected which might result in producing a dysfunctional protein. It is also important to note that two other known mutations at position 177 (Gly to Cys) and 298 (Phe to Cys) have been implicated to cause OI [8, 25]. The highly conserved glycine at 177 allow for a compact protein core while the mutated cysteine may disrupt this critical protein site [8]. WNT proteins are characterized by a set of 22 conserved cysteine residues that form the intra-chain disulfide bridges that maintain the tertiary protein structure [26]. This suggests that the mutations involving cysteine residues of WNT1 may have serious impact on the protein stability and function.

Mutations in *WNT1*have been associated with extreme phenotypes of osteoporosis, such as prenatally-onset severe OI, idiopathic juvenile osteoporosis and pregnancy-associated osteoporosis, confirming WNT1’s central role in regulation of bone strength. While biallelic mutations, like in our family, result in severe deforming OI, heterozygous mutations result in childhood-onset or early adulthood-onset osteoporosis with spinal and peripheral fractures, low BMD and low bone turnover, but normal growth and no deformities. Several mutations have been reported. Our patients with homozygous *WNT1* mutations had severe growth failure and early-onset skeletal fragility leading to severe deformities and loss of ambulation. This skeletal phenotype is consistent with other cases of OI caused by homozygous *WNT1* mutations [27]. No skeletal phenotype was evident in the heterozygous mutation carriers in our family, but no systematic phenotyping with BMD assessment was performed. None of the reported patients received bisphosphonate treatment and it remains unknown whether early-onset therapy would be as efficient as in type I collagen related OI in improving growth and preventing fractures and deformities.

## Conclusion

We report a novel mutation G324C in WNT1 gene responsible for severe recessively inherited *Osteogenesis Imperfecta*. This is being reported for the first time in Pakistan as well as globally. Our report expands the phenotypic and genotypic spectrum of WNT1-related OI. OI is a highly divergent disease with different inheritance patterns and variable severities and further studies on the clinical and functional consequences of different *WNT1* mutations may provide important insight into the roles of this gene in bone function and development. Optimal treatment remains to be established in future studies.

## Additional files

Additional file 1: **Table S1.** Clinical findings in the nine affected family members with a homozygous *WNT1* mutation. The “+” signs indicates the presence and severity of the symptom and “-” sign indicating the absence of the symptom. (DOCX 15 kb)

Additional file 2: **Table S2.** Identification of a single homozygous segment on chr12 overlapping between all cases and none of the controls. (DOCX 15 kb)

Additional file 3: **Table S3.** List of rare homozygous variants in III:5 and III:15 located within the shared homozygous segment on chr12. (DOCX 13 kb)

Additional file 4: **Figure S1.** Sanger sequencing results of affected family. Both parents (II:3 and II:4) (right upper and lower panels) of Patient 1 were heterozygous carriers, arrows indicate the double peaks (heterozygosity). Patient 1 (III:9) (left lower panel) result indicates homozygous change, arrow is indicating the change. While an unaffected individual (III:11) (left upper panel) showed both correct/wild type alleles. (DOCX 106 kb)
