# Correction to: Novel mutation G324C in *WNT1* mapped in a large Pakistani family with severe recessively inherited *Osteogenesis Imperfecta*

**DOI:** 10.1186/s12929-019-0525-x

**Published:** 2019-04-28

**Authors:** Mehran Kausar, Saima Siddiqi, Muhammad Yaqoob, Sajid Mansoor, Outi Makitie, Asif Mir, Chiea Chuen Khor, Jia Nee Foo, Mariam Anees

**Affiliations:** 1 Department of Biochemistry, Faculty of Biological Sciences, Quaid-i-AzamUniversity, University Road, Islamabad, 45320 Pakistan; 2 Institute of Biomedical and Genetic Engineering (IBGE) Islamabad, Islamabad, 44000 Pakistan; 3 Department of Genetics, Children Hospital, Lahore, Pakistan; 4 Atta-ur-Rehman School of Applied Biosciences, NUST, Islamabad, Pakistan; 5 Children’s Hospital, University of Helsinki and Helsinki University Hospital, Helsinki, Finland; 6 Folkhälsan Institute of Genetics, Helsinki, Finland; 7 Department of Bioinformatics & Biotechnology, Faculty of Basic and Applied Sciences, International Islamic University (IIU), H-10, Islamabad, 44000 Pakistan; 8 Human Genetics, A*STAR, Genome Institute of Singapore, Singapore, 138672 Singapore; 9 Lee Kong Chian School of Medicine, Nanyang Technological University, Singapore, 308232 Singapore; 10 Department of Microbiology, Faculty of Life Sciences, University of Central Punjab, Lahore, Pakistan

**Correction to: J Biomed Sci**

**https://doi.org/10.1186/s12929-018-0481-x**

In the original publication of this article [1], there are two errors in the article which the cDNA position of the pathogenic variant *WNT1* p.Gly324Cys should be c.970G>T instead of c.1168G>T.
