# Supplementary material for: Novel mutation G324C in WNT1 mapped in a large Pakistani family with severe recessively inherited Osteogenesis Imperfecta
Source: J Biomed Sci. 2018 Nov 17;25:82. doi: 10.1186/s12929-018-0481-x (PMC6240425; doi:10.1186/s12929-018-0481-x)
Supplement: Supplementary file 1 — Table S1. Clinical findings in the nine affected family members with a homozygous WNT1 mutation. The “+” signs indicates the presence and severity of the symptom and “-” sign indicating the absence of the symptom. (DOCX 15 kb) [file 12929_2018_481_MOESM1_ESM.docx]

**Additional file 1: Table S1.** Clinical findings in the nine affected family members with a homozygous *WNT1* mutation. The “+” signs indicates the presence and severity of the symptom and “-” sign indicating the absence of the symptom.

| **Affected Individual** | **III:1** | **III:3** | **III:5** | **III:9** | **III:13** | **III:14** | **III:15** | **IV:1** | **IV:2** |
| --- | --- | --- | --- | --- | --- | --- | --- | --- | --- |
| **Disease Severity** | Severe | Severe | Severe | Severe | Severe | Severe | Severe | Moderate | Moderate |
| **Age (Years)** | 23 | 25 | 16 | 11 | 20 | 17 | 10 | 14 | 12 |
| **Sex** | F | F | F | M | M | F | M | F | F |
| **Height (cm)** | 89 | 87 | 94 | 106 | 99 | 89 | 107 | 96 | 94 |
| **Weight(kg)** | 19 | 19.5 | 18 | 20 | 22 | 19 | 21 | 16 | 15.5 |
| **Severity of fractures** | ++ | + | + | + | + | ++ | +++ | + | + |
| **Mobility (Wheel chair bound)** | Yes | Yes | Yes | Yes | Yes | Yes | Yes | Yes | - |
| **Bowing of upper extremities** | +++ | +++ | ++ | + | ++ | ++ | ++++ | ++ | + |
| **Shortening of upper extremities** | ++ | ++ | + | + | + | + | ++ | + | + |
| **Shortening of lower extremities** | +++ | ++ | ++ | ++ | + | + | ++ | ++ | + |
| **Bowing of lower extremities** | ++ | ++ | + | + | + | + | ++ | + | + |
| **Hearing loss** | - | - | - | Mild | - | - | - | Mild | - |
| **Mildly blue sclerae** | Yes | Yes | - | Yes | - | Yes | - | Yes | - |
| **Dental Problems** | - | - | - | - | - | - | - | - | - |
| **Neurological Problems** | Mild | Mild | - | - | - | Mild | - | - | - |
| **Breathing Problems** | Mild | Mild | - | - | - | Mild | Mild | - | - |
| **Cardiac problem** | - | - | - | - | - | - | - | - | - |
