# Supplementary material for: Novel mutation G324C in WNT1 mapped in a large Pakistani family with severe recessively inherited Osteogenesis Imperfecta
Source: J Biomed Sci. 2018 Nov 17;25:82. doi: 10.1186/s12929-018-0481-x (PMC6240425; doi:10.1186/s12929-018-0481-x)
Supplement: Supplementary file 2 — Table S2. Identification of a single homozygous segment on chr12 overlapping between all cases and none of the controls. (DOCX 15 kb) [file 12929_2018_481_MOESM2_ESM.docx]

**Additional file 2: Table S2.** Identification of a single homozygous segment on chr12 overlapping between all cases and none of the controls.

| **Individual** | **Status** | **Chr** | **SNP1** | **SNP2** | **Start (hg19)** | **End (hg19)** | **Length KB** | **No. of SNPs** | **No. of matching segments** | **Allele group** |
| --- | --- | --- | --- | --- | --- | --- | --- | --- | --- | --- |
| III:13 | Case | 12 | rs2408141 | rs4533076 | 45,503,563 | 56,069,231 | 10,566 | 2060 | 9 | 1 |
| III:14 | Case | 12 | rs2408141 | rs4533076 | 45,503,563 | 56,069,231 | 10,566 | 2060 | 9 | 1 |
| III:15 | Case | 12 | rs2408141 | rs1894035 | 45,503,563 | 52,645,754 | 7,142 | 1280 | 9 | 1 |
| IV:1 | Case | 12 | rs2731032 | rs4533076 | 45,477,074 | 56,069,231 | 10,592 | 2062 | 9 | 1 |
| III:1 | Case | 12 | rs6582600 | rs4533076 | 37,942,622 | 56,069,231 | 18,127 | 3254 | 9 | 1 |
| IV:2 | Case | 12 | rs6582600 | rs4533076 | 37,942,622 | 56,069,231 | 18,127 | 3254 | 9 | 1 |
| III:3 | Case | 12 | rs6582600 | rs2701128 | 37,942,622 | 52,429,520 | 14,487 | 2401 | 9 | 1 |
| III:5 | Case | 12 | rs6582600 | rs2701128 | 37,942,622 | 52,429,520 | 14,487 | 2401 | 9 | 1 |
| III:9 | Case | 12 | rs6582600 | rs2701128 | 37,942,622 | 52,429,520 | 14,487 | 2401 | 9 | 1 |
| III:6 | Control | 12 | rs6582600 | rs7963439 | 37,942,622 | 46,084,699 | 8,142 | 1294 | 9 | 1 |
| III:12 | Control | 12 | rs6582600 | rs11829800 | 37,942,622 | 52,569,928 | 14,627 | 2443 | 0 | 2 |
| Consensus | | **12** | **-** | - | **46,084,699** | **52,429,520** | **6,344,821** | **-** | - | 1 |
