# Supplementary material for: Novel mutation G324C in WNT1 mapped in a large Pakistani family with severe recessively inherited Osteogenesis Imperfecta
Source: J Biomed Sci. 2018 Nov 17;25:82. doi: 10.1186/s12929-018-0481-x (PMC6240425; doi:10.1186/s12929-018-0481-x)
Supplement: Supplementary file 3 — Table S3. List of rare homozygous variants in III:5 and III:15 located within the shared homozygous segment on chr12. (DOCX 13 kb) [file 12929_2018_481_MOESM3_ESM.docx]

**Additional file 3: Table S3.** List of rare homozygous variants in III:5 and III:15 located within the shared homozygous segment on chr12.

| **Chr** | **Position** | **Ref** | **Alt** | **Gene Name** | **Amino Acid Substitution** | **SIFT Prediction** | **ExAC South Asians** | **ExAC Europeans** |
| --- | --- | --- | --- | --- | --- | --- | --- | --- |
| 12 | 48240233 | G | A | *VDR* | A303A | N/A | 0.194% | 0.532% |
| 12 | 49086943 | C | T | *CCNT1* | R685H | TOLERATED | 0.000% | 0.006% |
| 12 | 49375280 | G | T | *WNT1* | G324C | DAMAGING | Not found | Not found |
