# Supplementary material for: Novel mutation G324C in WNT1 mapped in a large Pakistani family with severe recessively inherited Osteogenesis Imperfecta
Source: J Biomed Sci. 2018 Nov 17;25:82. doi: 10.1186/s12929-018-0481-x (PMC6240425; doi:10.1186/s12929-018-0481-x)
Supplement: Supplementary file 4 — Figure S1. Sanger sequencing results of affected family. Both parents (II:3 and II:4) (right upper and lower panels) of Patient 1 were heterozygous carriers, arrows indicate the double peaks (heterozygosity). Patient 1 (III:9) (left lower panel) result indicates homozygous change, arrow is indicating the change. While an unaffected individual (III:11) (left upper panel) showed both correct/wild type alleles. (DOCX 106 kb) [file 12929_2018_481_MOESM4_ESM.docx]

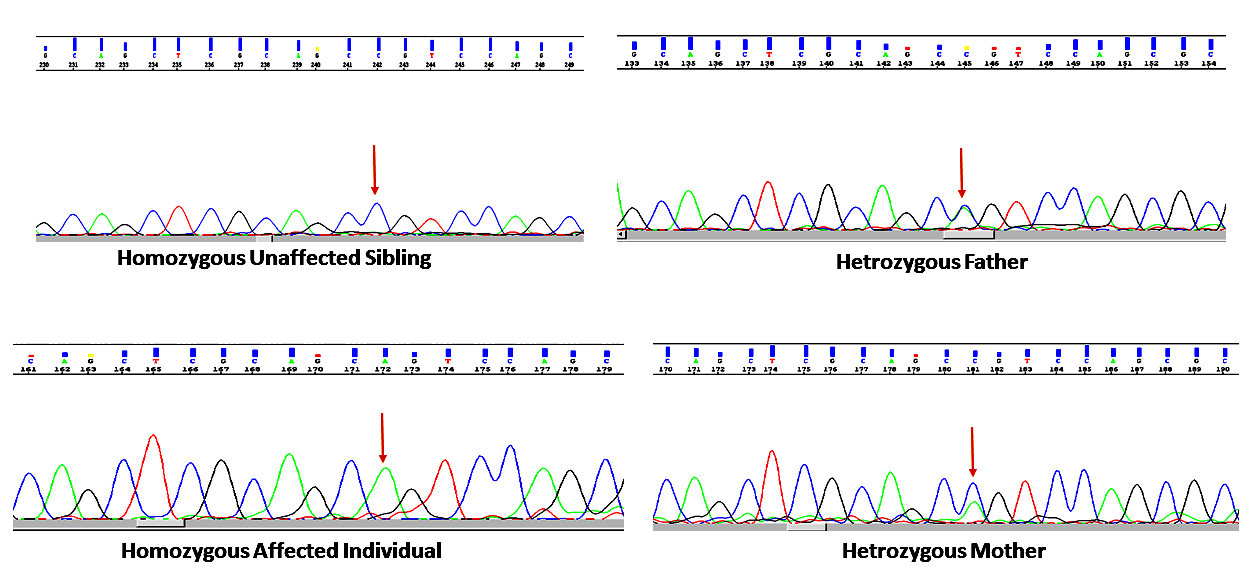


**Supplementary Figure S1.** Sanger sequencing results of affected family. Both parents (II:3 and II:4) (right upper and lower panels) of Patient 1 were heterozygous carriers, arrows indicate the double peaks (heterozygosity). Patient 1 (III:9) (left lower panel) result indicates homozygous change, arrow is indicating the change. While an unaffected individual (III:11) (left upper panel) showed both correct/ wild type alleles.
